# Chew the Pain Away: Oral Habits to Cope with Pain and Stress and to Stimulate Cognition

**DOI:** 10.1155/2015/149431

**Published:** 2015-05-18

**Authors:** Roxane Anthea Francesca Weijenberg, Frank Lobbezoo

**Affiliations:** ^1^Department of Clinical Neuropsychology, VU University Amsterdam, Van der Boechorststraat 1, 1081 BT Amsterdam, Netherlands; ^2^Department of Oral Kinesiology, Academic Centre for Dentistry Amsterdam (ACTA), University of Amsterdam and VU University Amsterdam, MOVE Research Institute Amsterdam, Gustav Mahlerlaan 3004, 1081 LA Amsterdam, Netherlands

## Abstract

The acute effects of chewing gum on cognitive performance, stress, and pain have been intensively studied in the last decade. The results have been contradicting, and replication studies proved challenging. Here, we review some of the recent findings of this topic and explore possible explanations for these discrepancies by incorporating knowledge derived from studies into oral habits and bruxism. Both stress and cerebral functional specialization (i.e., the involvement of specific brain structures in distinctive cognitive processes) are hypothesized to play a major role in the underlying physiological mechanisms of the diverse effects of chewing gum on cognition, stress, and pain.

## 1. Introduction

Mastication is essential for grinding our food into smaller particles [1]. During chewing, saliva is added to the particles to lubricate them and create a food bolus that can be swallowed [1]. Recently, reports have been published in the literature, stating that mastication might also serve other purposes, such as countering negative effects of stress [2] or aiding in cognitive function [3, 4]. It could be argued that the positive effects of mastication are similar to those of physical activity [5]. Although mastication is of course not the same as an intensive workout, it does have properties similar to exercise: it also increases heart rates [6, 7] and cerebral blood flow [8–10]. There are also direct cardiovascular improvements resulting from exercise [11]. Physical activity is a key element of an enriched environment, so while exercising, one is also experiencing an enriched environment [12], which has been shown to improve cognition [13]. Exercising can also attenuate the negative effects of stress [14]. Several studies have explored the relationship between mastication, cognition, stress, and pain. These studies will be discussed in more detail below. The aim of this review was to explore the relationship between mastication, cognition, and pain and to hypothesize on possible explanations.

## 2. Oral Habits

### 2.1. Gum Chewing

In two recent reviews [15, 16], the effects of chewing a piece of gum on cognition and stress in human volunteers are described. The outcomes of these reviews are summarized in Table 1. Although the papers have great overlap in the literature they included, the authors sometimes come to different conclusions. For example, while, in one paper, the authors emphasize that chewing gum cannot be seen as an aid for mental challenges [16], in the other paper, the authors conclude that chewing gum enhances alertness and that it might very well improve cognitive performance [15]. This discrepancy can partly be explained by the observation that Allen and Smith [15] are more lenient in their conclusions, as they view the majority vote as convincing, whereas Tucha and Koerts [16] focus on the contradictions and possible detrimental effects of chewing gum.

Nevertheless, both reviews agree that working memory is positively affected by chewing a piece of gum. It is interesting that both also agree in their conclusion that divided attention is unaffected. Some earlier papers mentioned that the distracting (novelty) effect of chewing gum while performing a task might have influenced test results, but the fact that divided attention is not affected argues against this.

As there are still many unexplained and contradictory findings, a final conclusion cannot yet be made on the acute effects of chewing a piece of gum on cognitive performance and stress. Underlying physiological mechanisms remain to be identified, and the time on task might be of influence, for example, how long participants chewed and whether they chewed only prior to examination or also during the test. It is possible that chewing gum has a transient positive effect, but only after cessation of chewing, since chewing while on task can have a negative effect [15, 16]. It would be interesting to see what effect other oral habits have on stress and cognition.

### 2.2. Bruxism

In a sample of children (7–17 years), with attention deficit hyperactivity disorder (ADHD), it was observed that levels of oral habits such as nail or pencil biting and bruxism were all elevated [17]. Bruxism is “a repetitive jaw-muscle activity characterized by clenching or grinding of the teeth and/or by bracing or thrusting of the mandible” [18]. The activity is involuntary and can occur during sleep (sleep bruxism) or during waking moments (awake bruxism) [19]. Nail biting is an oral habit that is commonly seen in the general population, and triggers for it are, amongst others, anxiety, stress [20], boredom, and frustration [21]. Anxiety, stress, boredom, and frustration are also the most common triggers for other (pathological) body-focused repetitive behaviors, such as skin picking and hair pulling [22]. The need that is being met by engaging in these self-damaging behaviors is thought to be “relief from negative affective states” [22].

Those suffering from their habits, for example, bruxers with complaints of pain or other temporomandibular disorders (TMD), usually seek the help from their dentist rather than a psychologist [23]. This is perhaps reflected in the current popular treatment options for bruxism: it is typically treated with oral splints, which is reflected in a publication bias with regard to bruxism therapies [23]. Behavioral therapies are the least popular therapies to be scientifically explored, while pharmaceutical approaches have been gaining popularity [23]. Current therapies seem to be focused on preventing damage, rather than finding and treating the cause [24]. This is most unfortunate, of course, as options for treatments are disregarded this way. Risk factors for bruxism were defined as peripheral, such as malocclusion, or central (pathophysiological or psychosocial) [25, 26]. It was concluded that peripheral causes are not likely to play a significant role in the etiology of bruxism [19, 25–27]. It can be argued that bruxism is a disorder from the central neurotransmitter system since the basal ganglia (part of the extrapyramidal system) and the thalamic pathways are implicated in the origin of bruxism, with a crucial role to play by the neurotransmitter dopamine [25]. Experienced stress plays a mediating role, at least in awake bruxism [28], while others argue that stress might even be the main cause for bruxism [27].

This latter theory is fitting with the observations that experienced daily life stress is related to daytime clenching [29] and that self-reported bruxers have higher anxiety levels and more often experience severe stress compared to healthy controls [30].

## 3. Mastication, Stress, and Pain

### 3.1. Mastication and Stress Relief

Chewing and clenching have been implied as a way to relieve stress and provide relaxation [31]. The chewing force needed to chew a piece of gum correlated to the amount of salivary cortisol reduction after performing a stressful task [32]. In restrained rats, the length of stress-induced bruxism activity correlated inversely to physiological parameters of stress, such as blood cortisol and adrenaline levels [33]. Whether this stress relieving effect is robust over prolonged periods of time is not yet clear. A longitudinal study examined the effect of regular gum chewing during 14 days (leisurely chewing a piece of gum for at least 5 minutes, twice per day) in young adults [34]. After these two weeks, chewing gum was associated with decreased scores for anxiety, depression, fatigue, and confusion compared to a control group. This benefit of chewing was transient; however, as after 4 weeks (i.e., two weeks after stopping with the intervention), there was no longer a difference between the groups [34]. Another experiment showed a transient cerebral response to changes in the masticatory domain: using fMRI, it was shown that after being fitted with a new dental prosthesis, adaptive brain activation in the right and the left precentral and postcentral gyrus occurred during oromotor tasks like jaw clenching, but only in the first three months, even though the prosthesis was worn continuously [35].

#### 3.1.1. Salivary Cortisol

Some comments with regard to cortisol assessments need to be made. There are two types of human cortisol that can be assessed: total cortisol and free cortisol. The latter can be sampled in blood, urine, or saliva [36]. Levine and colleagues [36] emphasize that despite current popularity of these “biomarkers of stress,” the question remains unanswered whether these assessments reflect actual metabolic and cerebral functioning, namely, activity of the hypothalamic-pituitary-adrenal (HPA) axis, as the hypotheses on which these assessments rely are, in fact, still hypotheses [36]. Salivary cortisol assessments can provide some useful information, but the sampling technique is prone to false results, for example, due to pH changes after eating or drinking or due to contamination by blood from oral lesions [36]. Furthermore, only small correlations have been found between salivary cortisol and plasma free cortisol measurements (the latter being the gold standard) [36]. In another comprehensive review discussing salivary cortisol, it is shown that there is in fact little scientific support for the popularly assumed correlation between psychological stress and the endocrine response [37]. Hellhammer and colleagues [37] emphasize that although salivary cortisol can be used as a biomarker for perceived stress, this can only be done with great caution, and one must be aware that there will only be a moderate association with perceived, or task-induced, stress [37]. The salivary cortisol response is influenced by many factors, such as estrogens (gender, menstrual phase, and oral contraceptives), certain drugs, presence of chronic stress, long-term exercise [37], and overall physical fitness [11]. Sleep and the circadian rhythm also influence the HPA axis activity [38, 39] and finally one must keep in mind that cortisol levels also are not stable during the waking hours but exhibit ultradian oscillations [39]. Clearly, reports relying on salivary cortisol assessments have to be viewed with some caution.

### 3.2. Mastication and Pain

It is known that, in newborn babies, rhythmic oral motions, such as during breastfeeding or sucking on a pacifier, but also sweet taste such as that from breast milk, or glucose or sucrose solutions, are nonpharmacological approaches for pain relief [40]. Building on this knowledge, it was investigated whether sweet taste, chewing gum, or a combination of both could relieve pain in 7–12-year-old children, undergoing venipuncture or vaccination [41]. The authors did not find an overall effect of the intervention on pain responses. In boys, continuously chewing unsweetened gum reduced pain scores and ratings of unpleasantness. In girls, however, the opposite was observed: chewing sweetened gum reduced pain scores, but chewing unsweetened gum increased them [41]. It should be added that since the control group was also given chewing gum, prior to the procedure, it is possible that some carry-over effect of this chewing took place. On the other hand, the children chewed gum for only a brief period: the control group chewed for 1 minute, and the intervention group chewed for 2 minutes and during the short medical procedure. It is possible that the overall time was too brief to evoke a strong response to chewing.

In another experiment to investigate the effect of chewing on pain and possible underlying neural mechanisms, participants were submitted to nociceptive flexion reflex (NFR) protocol [42]. The NFR protocol encompasses a painful electrocutaneous stimulation of the lower leg, after which the muscle activity in the upper leg on the same side is measured [43]. Blood samples were taken and brain perfusion was assessed with near-infrared spectroscopy measuring (de)oxygenated hemoglobin [42]. The subjects chewed a piece of mint-flavored gum leisurely for 20 minutes. Assessments (applying the NFR and taking blood samples) were made at baseline, immediately after chewing and 30 minutes after chewing. Gum chewing decreased the NFR both immediately after and 30 minutes after chewing [42]. Serotonergic blood levels were increased after chewing, and significant cerebral perfusion was increased in the ventral part of prefrontal cortex (PFC) [42]. The authors conclude that chewing a piece of gum apparently has analgesic effects, with the PFC mediating this effect through serotonergic neurons of the dorsal raphe nucleus [42]. The same dorsal raphe nucleus is implicated in the origin of disordered eating [44], in a hypothesis that by changing the eating behavior (e.g., adhering to a food-restricted diet) one also changes the serotonergic pathways between the PFC and dorsal raphe nucleus and thus alters mood. This theory seems to fit with the other observations that stimulation through chewing might have beneficial effects on affect.

In an animal experimental study, rats were fed a soft diet for 11 days [45]. Subsequently, they were injected in one of the paws with complete Freund's adjuvant (CFA) to temporarily increase their sensitivity to pain (hyperalgesia). In the following 3–6 days, they were fed hard food (intervention) or continued on soft food (control). Then, a heat stimulus was separately applied to both paws, and the reaction time (withdrawal latency) was measured. The difference in reaction speed between the injected and the control paw was taken as a measure for the induced hyperalgesia. Rats on the hard diet showed less CFA-induced hyperalgesia. This protective effect of hard food was gone after injection with the opioid-antagonist naloxone [45]. Hard food was also protective against inflammation, as was shown by a decrease in activity in immunoreactive cells. Inhibiting sensory pathways, by cutting the inferior alveolar nerve or removing the primary somatosensory cortex, reduced but not completely reversed this effect [45]. The authors conclude that a protective effect from hard food might involve the opioid system, which is affected by sensory pathways, but perhaps also by other neural pathways, such as the brainstem reticular formation [45].

A coupling of the endogenous opioid system and the stress response has been widely studied and is well established [46, 47]. For example, it was found that endogenous opioid systems attenuate the stress response in pregnancy [48]. Underlying mechanisms are being studied: the attenuating effect of suppression of kappa-opioid receptors on the stress response has been shown in a wide range of animal models [46], and endogenous opioids were found to negate the detrimental effects of stress hormones by protecting the endothelial function, a condition which is thought to underlie cardiovascular disease [49]. The locus coeruleus is thought to play a key role in the interaction of the opioid system and the stress response [47].

## 4. Discussion

The literature discussed above has shown that there currently is an interest in the relationship between mastication, cognition, stress, and pain. The acute effects of chewing gum on cognitive measures and biomarkers of stress, such as salivary cortisol, have not yet generated univocal results. However, a closer examination of cerebral functional specialization of cognitive functions might provide insight into the ambiguous results that are currently being reported.

### 4.1. Cerebral Functional Specialization

The subcortical basal ganglia are hypothesized to play a role in bruxism [25]. Interestingly, they are also involved in cognitive functions, such as the nondeclarative memory (also known as the procedural memory) [50]. Basal ganglia are also known to play a role in habit learning, most notably through feedback based learning (rewards and/or punishment) regardless of whether this learning is implicit or explicit [51]. This concurs with the finding that chewing a piece of gum can enhance working memory [52], which was assessed by having participants performing a routine that had to become habituated, and the finding that spatial memory (learning the route to an escape platform) was impaired in mice by removing the upper molar crowns [53].

The working memory enhancement of chewing gum would indicate a positive stimulus of chewing for the PFC and basal ganglia [54] and also for the medial temporal lobe and hippocampus for longer tasks [55]. Interestingly, short-term working memory function was not negatively impacted by physiological stress, but it was observed that task difficulty (which could be considered psychological stress) negatively influenced performance [56]. Participants showed elevated levels of activation in the PFC while maintaining their performance levels, a cerebral response which is thought to compensate for the distraction of the stressor [56]. If chewing reduces stress, this would facilitate the compensatory process and thus positively affect working memory performance. Both the hippocampus and the PFC are known for their sensitivity to stress [57], and a reduction of stress due to chewing would explain the positive effect of gum on the behavioral outcomes.

Declarative memory function (which can be subdivided into episodic/autobiographic and semantic memory) is involved in recall and recognition tasks [50]. This type of memory is localized throughout the brain, including the medial and temporal lobes [51], but key areas for recall memory are the frontal and parietal lobes and the cerebellum, while learning and storing new memories (i.e., reproduction and recognition) involve the hippocampus and the parahippocampal gyrus [50]. The fact that these kinds of memory are not enhanced by chewing a piece of gum is therefore perhaps not surprising, as they do not rely primarily on the basal ganglia. Most of the studies included in the two reviews discussed here [15, 16] did not use giving feedback (punishment or rewards) in their paradigm, and thus, they did not activate the basal ganglia [51]. Recently, Bos et al. showed that recall and recognition of newly learned words were improved in a stress condition (i.e., putting the hand in painfully cold water) [58]. This is also fitting with the current observations of the effects of chewing gum: if chewing indeed reduces stress, this would also negate any stress-induced enhancement of the long-term memory.

The lack or even detrimental effect of chewing on vigilance is most likely a negative effect on the vigilance network and the ascending reticular activating system (ARAS) [59]. The ARAS has also been suggested to play a role in sleep bruxism: minutes prior to the onset of sleep bruxism, arousal responses such as increased heart rate and muscle tone are observed in bruxers, which are indicators of ARAS activation [60]. It might be possible that active mastication reverses the same pathway and thus downregulates the ARAS, causing the negative effect on vigilance. Others reported that sleep bruxers do not perform better or worse than controls in neuropsychological tests for vigilance or motor response [61], which is fitting with the current observations.

## 5. Conclusion

Mastication, as other physical activities, can most likely relieve (acute) stress and even pain. Bruxers and nail biters might unknowingly draw upon this effect, in order to alleviate their commonly reported anxiety. Active mastication might improve some measures of cognitive performance, such as working memory [15, 16] or subjective alertness [15]. Bruxers have not been shown to display cognitive advantages over nonbruxers, with regard to vigilance [61]. This is perhaps not surprising, as chewing has not been shown to enhance vigilance either and, in fact, negatively effects it in children [62]. It would be interesting to see if bruxers outperform nonbruxers in other cognitive domains, such as spatial or working memory. Treatment for bruxism typically shows a dental focus, offering splints or other occlusal appliances. Counseling by a psychologist and a physical therapist in order to learn relaxation can be complementary to this [23].

Other populations that might benefit from these insights are persons at risk for cognitive decline or mental instability, such as older persons suffering from dementia and psychiatric patients. They might experience positive effects from oral and dental care and eating a diet that consists of hard, chewing-enhancing foods. Maintenance of masticatory function should be endeavored for all clinical groups. However, the long-term use of chewing gum and engaging in other habits should not be encouraged, as this increases the risk for complaints of fatigue, tenderness, and even pain in the musculoskeletal structures of the masticatory system [63–67].

## Highlights

- The heterogeneous effects of chewing gum on cognitive performance can partially be explained by cerebral functional specialization and the involvement of the basal ganglia.
- Stress and relief of stress can play an important role in the physiological mechanisms underlying these effects.
- Oral habits such as bruxism might draw upon the same effects for stress relief.
- Active chewing might relieve stress or pain, but long-term engagement in oral habits increases the risk of fatigue, pain, or temporomandibular disorders.

## Figures and Tables

**Table 1:** The outcomes of two reviews on the effects of mastication on cognition and stress in healthy volunteers.

Variable	Allen and Smith, 2011 [15]	Tucha and Koerts, 2012 [16]
*Cognitive outcomes *		
Academic performance		+
Alertness		
Subjective	+	
Attention		
Divided	0	0
Selective	±	±
Shifting		±
Sustained/vigilance	±	±
Executive functioning		0
Memory		
Context dependent	0	±
Recall	±	±
Recognition	−	
Working	+	+
Test performance		−
Speed	+	±
Spatial skill		±
*Stress related outcomes *		
Biomarkers (i.e., pupil dilation, heart rate)	±	
Acute, self-reported	0	
Chronic, self-reported	+	
Salivary cortisol	±	

The outcomes of two reviews on mastication, cognition, and stress. + = the authors report a positive effect; − = the authors report a negative effect; 0 = the authors report no effect; ± = the authors report contradicting results in the literature.
